# Congenital Descending Aorta-Right Atrial Tunnel: A Case Report

**DOI:** 10.3389/fped.2020.00524

**Published:** 2020-09-08

**Authors:** Xing Zhang, Zhongjian Su, Yanfei Yang, Liping Ge, Bin Li

**Affiliations:** Department of Cardiology, Kunming Children's Hospital, Kunming, China

**Keywords:** congenital heart defects, computed tomography angiography, transcatheter closure, ventricular septal defect occluder, congenital descending aorta-right atrial tunnel

## Abstract

**Introduction:** Congenital descending aorta-right atrial tunnel is a rare congenital heart defect. Herein, a new case successfully treated by transcatheter closure using a new type of ventricular septal defect occluder from the aortic side was reported.

**Case Presentation:** An 11-month-old Chinese girl presenting with a cardiac murmur was suspected with partial anomalous pulmonary venous connection as assessed by echocardiography. Descending aorta-right atrial tunnel was confirmed by computed tomography angiography and cardiac catheterization. Subsequently, transcatheter closure was performed successfully using a new type of ventricular septal defect occluder from the aortic side. The cardiac murmur disappeared after the intervention, and echocardiography did not reveal any abnormal flow inside the right atrium. At 6 months, the patient had no murmur, and no residual shunt was found using the echocardiogram.

**Conclusion:** Descending aorta-right atrial tunnel is a rare anomaly. Transcatheter closure was successful in our case. Long-term follow-up is needed to assess any progressive growth of the residual tunnel.

## Introduction

Congenital aorta-right atrial tunnel (ARAT) is a rare congenital heart defect which has been reported previously. It is characterized by an abnormal tunnel-like communication between the aorta and the right atrium. This tunnel originates typically from the aortic sinus, and rarely from the descending aorta (1, 2). ARAT can be treated with surgery or by a transcatheter device placement using an Amplatzer duct occluder or Amplatzer duct occluder II from the right atrium (3). Here, we present a case of a descending aorta-right atrial tunnel that was successfully closed with a new type of ventricular septal defect (VSD) occluder from the aortic side.

## Case Description

**Figure 1:**
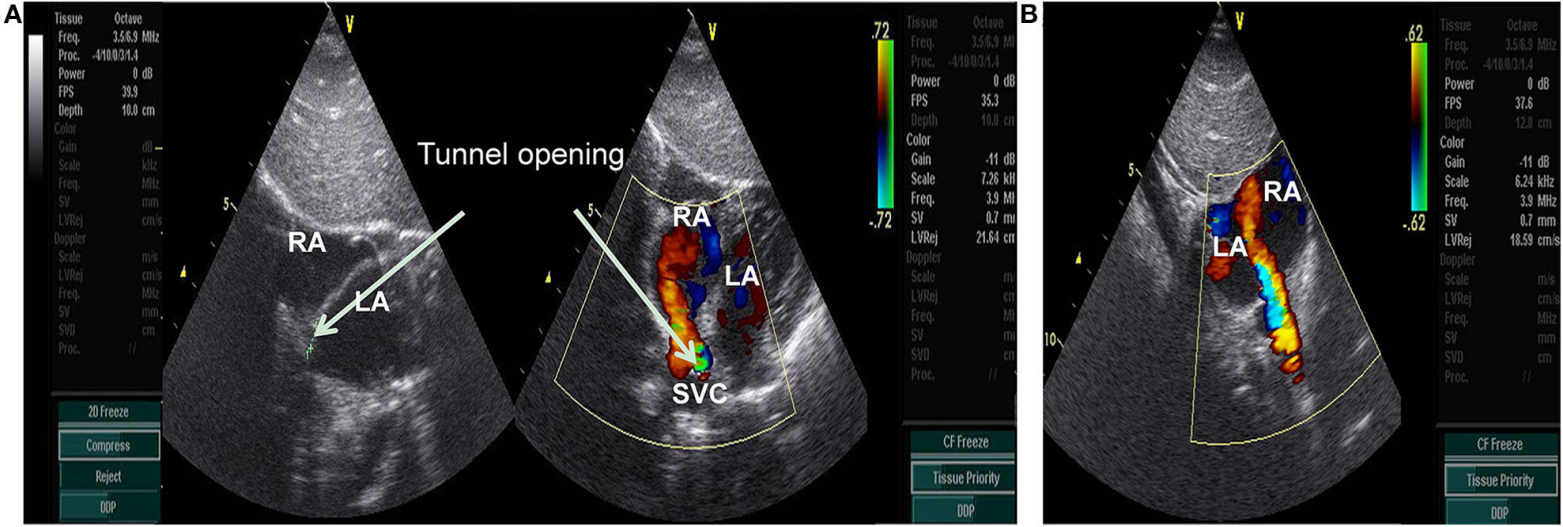
**(A)** subcostal 2-chamber view: Abnormal color flow was observed at the junction of the superior vena cava and the right atrium, showing a continuous spectrum(arrow). RA, right atrium; LA, left atrium; SVC, superior vena cava. **(B)** subcostal 2-chamber view postoperative: Abnormal color flow was disappeared at the junction of the superior vena cava and the right atrium.

**Figure 2:**
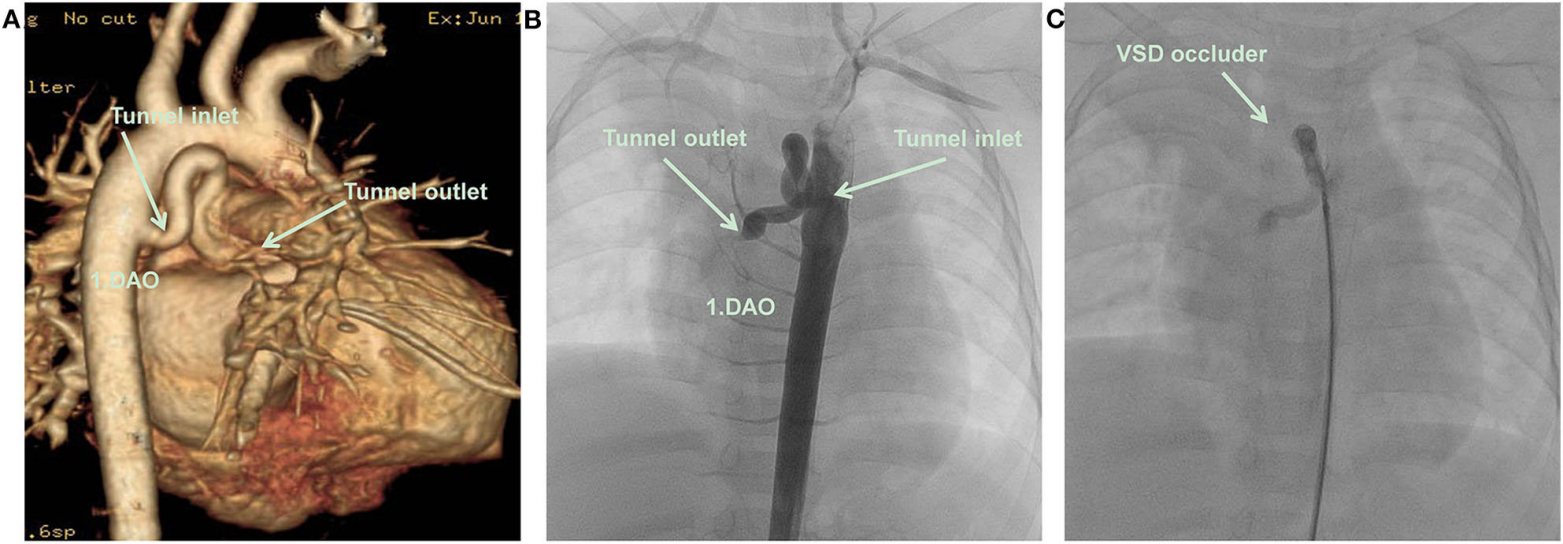
**(A)** CTA: An abnormal blood vessel developed from descending aorta, and zigzagged to the right atrium. **(B)** Cardiac catheterization: An abnormal blood vessel developed from descending aorta, and zigzagged to the right atrium. **(C)** A VSD occluder has been placed at the inlet of tunnel, there was no obvious shunt revealed by aortography after closure. DAO, descending aorta; RA, right atrium; VSD occluder, ventricular septal defect occluder.

A 9.2 kg 11-month-old Chinese girl was admitted to the Kunming Children's Hospital for a cardiac murmur. The parents of the child reported no symptoms or growth retardation, and there was no family history of congenital heart defects. A 2/6 systolic murmur was heard at the upper right sternal border. A routine echocardiography was performed, and a diagnosis of partial anomalous pulmonary venous connection was suspected (Figure 1A). Subsequently, computerized tomography angiography (CTA) demonstrated an abnormal blood vessel from the descending aorta, ascending across the left main bronchus, and then descending to end in the right atrium (Figure 2A).

## Diagnostic Assessment

The patient underwent cardiac catheterization. The right femoral artery was cannulated using 5 Fr sheaths, and the patient was heparinized (100 U/kg). Aortography in the descending aorta demonstrated that an abnormal blood vessel was present from the right anterior wall of the descending aorta at the height equal to that of the 4–5th thoracic vertebrae and followed a tortuous course to the right atrium. The diameter of the tunnel inlet was 4 mm and that of the opening into the right atrium was only 1 mm (Figure 2B). An occlusion was performed using a 5 mm (waist diameter 5 mm, length 5 mm) new VSD occluder (Starway Medical Technology, Beijing, China) from the arterial side using a 4 Fr long sheath (Starway Medical Technology).

A 260 cm 0.032-inch super smooth guidewire (Starway Medical Technology) was inserted from the descending aorta side in the direction of blood flow to the distal tunnel. A 4 Fr Cobra catheter was guided into the tunnel, and the smooth guidewire was withdrawn and replaced with a 260 cm 0.035-inch stiff guidewire (Starway Medical Technology) to establish a tunnel-descending femoral orbital artery in the right aorta. The 4 Fr long sheath was inserted along the stiff guidewire. A 5 mm new VSD occluder was loaded, the distal disc of the occluder was fixed at the U-shaped bend of the tunnel, and the waist and proximal disc were fixed in the ascending branch of the U-shaped bend of the tunnel. No obvious shunt was revealed by aortography after device placement (Figure 2C). No procedural complications occurred and neither an antiplatelet nor antibiotic treatment was used after the procedure. An abnormal color flow was not seen on the postprocedural cardiac echocardiography. At 6 months of follow-up, the patient remained well, and no residual shunt was found using the echocardiogram (Figure 1B). The patient's parents provided informed consent for the patient's information including supporting images to be published.

## Discussion

Aorta-right atrial tunnel was first reported by Coto et al. (4), and it has been classified into two categories according to the original location. The left aortic sinus with a posterior extension is the origin for the majority, followed by the right aortic sinus with an anterior extension. The non-coronary aortic sinus is a rare origin (1). The terminal part of the tunnel is in the right atrium, which can be at the junction of the superior vena cava and on the right atrial cavity. This is often associated with secundum atrial septal defect and patent ductus arteriosus (PDA) (5). The tunnel originating from the descending aorta is also rare; hitherto, only two cases have been reported in 2003 (5) and 2017 (2). These included a 3-year-old girl with a cardiac murmur and a 4-day-old male infant with cardiac shock, respectively. Including our case, all three were infants with the tunnel originating from the descending aorta close to the PDA or the ligamentum arteriosum. The tunnel was tortuous with a restrictive outlet in the right atrium, especially in our case, and the outlet of the tunnel was narrow such that the cardiac murmur was muted.

We propose that ARAT be classified into two types: Type I: origin from the ascending aorta, further classified into Type Ia origin from the ascending aorta and extending anteriorly and Type Ib origin from the ascending aorta and extending posteriorly; Type II: the tunnel arises from the descending aorta.

Presently, the mechanism of ARAT formation is unclear. According to the histopathological examination of the tunnel, the characteristics are similar to those of the aorta. It is speculated that due to the abnormal development and the weakened support force of the aortic medial elastic fiber, the blood vessel wall gradually expands to form a capsule or a tube under the high pressure of the aorta, and sprouts into the right atrium because it is adjacent to the right atrium (6). However, the tunnel originating from the descending aorta in our case was distal from the right atrium, which cannot be explained by this theory; hence, we speculated it to be a variant of the aorta-pulmonary collateral arteries.

The pathophysiological manifestations of ARAT are similar to those of congenital heart diseases with a left-to-right shunt at the atrial level, dilated right atrium and right ventricle, and increased pulmonary blood flow. Clinical symptoms include fatigue, palpitation, dyspnea, pneumonia, and shock. Patients with a small shunt volume might not exhibit clinical symptoms. The findings of the physical examination primarily include continuous or systolic murmurs at the right sternal border (7).

ARAT is initially suggested by echocardiography and further confirmed by CTA or cardiac catheterization. For the tunnel originating from the descending aorta, it should be distinguished from PDA, aortopulmonary collateral arteries, and partial anomalous pulmonary venous connection.

Whether asymptomatic ARAT can be cured, is still controversial. Chandra et al. (8) suggested surgery owing to the risk of volume overload in the right heart, infective endocarditis, and tumor rupture. Lee et al. (5) recommended follow-up observation since the risk of surgery in asymptomatic ARAT patients outweighed the benefit.

Immediate treatment after diagnosis is recommended for symptomatic ARAT (1, 9). This treatment could be surgery or transcatheter closure. In surgery, the tunnel inlet and outlet are sutured, respectively, using a ligation or patch.

Transcatheter closure is an alternative if the tunnel is not extremely large, and embolizing the inlet or outlet is optional. Previously, embolizing the tunnel outlet from the right atrium side has been prevalent (2, 3, 7, 8), because a large occluder can be placed into the tunnel from the femoral vein with minimum vascular damage. In our case, embolizing the inlet was preferred owing to the stenosis of the outlet. In addition, the new VSD occluder can be delivered using only a 4 Fr or 5 Fr long sheath, which reduces the damage to the femoral artery. During the procedure, the disc of the occluder was placed in the tunnel to prevent the end of the occluder from protruding into the descending aorta, which in turn might cause descending aortic stenosis or hemolysis.

The main concern of transcatheter closure of the tunnel is thrombosis in the residual tunnel and residual tunnel expansion. Embolizing the outlet of the tunnel is conducted from the right atrium side with a slow blood flow into the aortic end of the tunnel; thereby, causing a turbulent flow which poses a high risk for thrombosis and whether the residual tunnel continues to expand is yet to be observed. Furthermore, no thrombosis in the tunnel and no residual tunnel expansion has been reported at 1-year follow-up in a previous case (1). However, the long-term prognosis still needs follow-up. Embolizing the tunnel inlet is conducted from the aortic side, which eliminates the possibility of expansion in the late stage, just like transcatheter closure of major aorta-pulmonary collateral arteries (MAPCAs) that are occluded in the middle portion by coiling. Typically they don't expand or rupture in the proximal course. Thus, we recommend embolizing the tunnel inlet from the aortic side.

Currently, there is no consensus on the use of antiplatelet therapy after the procedure. No abnormalities were observed during the follow-up in the cases of transcatheter closure from the right atrium side with or without antiplatelet drugs. In our case, no antiplatelet therapy was administered, and no abnormalities were observed at the 6 month follow-up.

Descending aorta-right atrial tunnel can be confirmed by CTA or cardiac catheterization, and is easily misdiagnosed as aorta-pulmonary collateral arteries. The transcatheter closing inlet of the tunnel using the new type of VSD occluder might be an optimal alternative to surgery.

## Data Availability Statement

The original contributions presented in the study are included in the article/supplementary material, further inquiries can be directed to the corresponding author/s.

## Ethics Statement

The studies involving human participants were reviewed and approved by Kunming Children's Hospital. Written informed consent to participate in this study was provided by the participants' legal guardian/next of kin. Written informed consent was obtained from the individual(s), and minor(s)' legal guardian/next of kin, for the publication of any potentially identifiable images or data included in this article.

## Consent for publication

Informed written consent was obtained from the patient for publication of this case report.

## Author Contributions

XZ, ZS, and BL carried out the studies, participated in collecting data, and drafted the manuscript and helped to draft the manuscript. YY and LG performed the statistical analysis and participated in its design. All authors contributed to the article and approved the submitted version.

## Conflict of Interest

The authors declare that the research was conducted in the absence of any commercial or financial relationships that could be construed as a potential conflict of interest.

## Abbreviations

ARAT: Aorta-Right Atrial Tunnel
CTA: Computerized Tomography Angiography
RA: Right Atrium
VSD: Ventricular Septal Defect.
